# Aortic Valve Replacement in the Failing Left Ventricle: Worthwhile?

**DOI:** 10.31083/j.rcm2307223

**Published:** 2022-06-24

**Authors:** Asanish Kalyanasundaram, Thais Faggion Vinholo, Mohammad A. Zafar, Osama Anis, Paris Charilaou, Bulat Ziganshin, John A. Elefteriades

**Affiliations:** ^1^Aortic Institute at Yale-New Haven Hospital, New Haven, CT 06510, USA; ^2^Saint Peter’s University Hospital, New Brunswick, NJ 08901, USA; ^3^Department of Cardiovascular and Endovascular Surgery, Kazan State Medical University, 420012 Kazan, Russia

**Keywords:** aortic valve replacement, ejection fraction, heart failure, intra-aortic balloon pump, aortic stenosis, aortic regurgitation

## Abstract

**Purpose:**

According to the 2020 American College of Cardiology/American Heart Association guidelines, the aortic valve should be replaced in the setting of severe aortic stenosis or regurgitation, independent of left ventricular function (even for EF <55%). However, in clinical practice, especially in a very low EF range, surgeons may avoid surgical aortic valve replacement (SAVR) because of concern over operative risk. This study examines outcomes of patients with EF ≤35% undergoing SAVR.

**Methods:**

From 2004 to 2019, 895 patients underwent SAVR for aortic stenosis (AS) and/or regurgitation (AR) by a single surgeon at our institution. From among these, 40 patients (4.47%) had an ejection fraction (EF) of 35% or less, forming the study group. Intra-aortic balloon pump was placed intraoperatively prophylactically pre-bypass in 18 out of the 40. Preoperative and post-operative echocardiograms were compared to determine changes in ejection fraction. Mid-term survival was assessed.

**Results:**

16 patients presented with AS, 20 with AR, and 4 with a combination of AS and AR. Hospital survival was 97.5% (one patient death). The average ejection fraction progressively improved over time from 26% initially to 46% mid-term with mean follow-up of 43 months (0.1–140.7). Remarkably, five-year survival was comparable between the study group and an age- and gender-matched general population (*p* = 0.834). Downward trends in LV end-diastolic diameter and end-systolic diameter were seen. The former achieved statistical significance (6.0 cm to 5.3 cm; *p* = 0.0046), while the latter fell slightly short (4.8 cm to 4.1 cm; *p* = 0.056). Patients in whom an IABP was used had lower EFs than those without IABP (range 10–35, mean 23% vs. 15–35%, mean 27.6%). The EFs of the three subgroups improved significantly postoperatively (*p *< 0.001 for AS, *p* = 0.002 for AR, and *p* = 0.046 for AS and AR).

**Conclusions:**

Surgical AVR can be done safely in patients with a failing LV with EF ≤35%. Significant improvements in the ejection fraction are seen over time. We believe there is a role for prophylactic pre-bypass IABP. Five-year survival is normalized. Surgeons should not hesitate to perform AVR in these highly jeopardized patients.

## 1. Introduction

ACC/AHA Practice Guidelines recommend aortic valve replacement surgery (SAVR) for patients with severe asymptomatic aortic regurgitation (AR) and a low left ventricular ejection fraction (EF ≤55%) and for severe symptomatic AR regardless of EF. AVR is also recommended for patients with severe asymptomatic aortic stenosis (AS) with a low EF (<50%) and all symptomatic severe AS patients (class I recommendations) [1]. In the 2000s and early 2010s, there was a clear reluctance by surgeons to perform SAVR in these patient populations, especially in a very low EF range. The Euro Heart Survey revealed that only 21.8% of patients with AR and a left ventricular ejection fraction (LVEF) between 30% to 50% received surgical intervention, dropping to 2.7% of patients with an EF <30%. These percentages were 16.4% and 2.9% respectively for AS [2]. This hesitancy may have stemmed from the observable association between lower preoperative LVEF and higher general postoperative mortality [3].

Despite subsequent advancements in the medical and surgical landscape, AVR is still likely underutilized. A recent report from 2022, for example, showed that in over 6000 AS patients for whom it was indicated or potentially indicated, only 48% of patients had an AVR [4]. Importantly, a low EF (<50%) was found to be a significant predictor of patients not receiving an AVR. The absolute number of low EF patients receiving an AVR has generally increased over the past several years, though this is largely owing to an increase in AS prevalence, which tracks with the aging population; the proportion of symptomatic cases receiving an AVR has reportedly remained steady since 2001. The rise in interventions is driven by TAVR, while the use of SAVR has generally declined in relative and absolute terms [4]. TAVR already makes up the majority of AVRs, including in over 80% of severe AS cases in Germany that were treated procedurally [5]. The availability of analogous data for AR is more limited, but one large survey published in 2019 showed that only 3.3% of patients who received an AVR had an EF <30% [6].

The safety and efficacy of TAVR has been convincingly demonstrated. Several recent studies looking at TAVR in low-EF patients show marked improvements in mortality and in EF [7, 8, 9]. The mortality and improvements in EF may be non-inferior to patients with a preserved or milder reduction in LVEF [10, 11]. The question addressed by this study, however, is whether a low EF in and of itself poses a risk significant enough to recommend against SAVR specifically, or whether these patients may still see significant clinical benefit. For young patients with low EF, for example, a durable mechanical valve may be quite strongly preferable to a biological TAVR valve.

We present here our single-center, single-surgeon retrospective analysis of a group of 40 very low EF patients with AS and/or AR who underwent SAVR and were followed up for several months to years.

## 2. Methods

### 2.1 Study Population

This is a retrospective cohort study from a large university medical center (Yale Aortic Institute, Yale New Haven Hospital, New Haven, Connecticut, USA). Using a database containing records from 2004 to 2019 of patients treated at the Yale Aortic Institute, we searched for patients who underwent SAVR for aortic stenosis and/or aortic regurgitation by a single surgeon (JE). 895 patients fit these criteria. Of these, 40 patients had an ejection fraction of 35% or less, and these made up our study population. The AHA/ACC diagnostic criteria for severe aortic stenosis include an aortic valve maximum velocity >4 m/s, a mean aortic transvalvular pressure gradient >40 mm Hg, or an aortic valve area <1.0 ${\mathrm{cm}}^{2}$. The diagnostic criteria for severe aortic regurgitation included a doppler jet width of ≥65% of the left ventricular outflow tract (LVOT), vena contracta >0.6 cm, or a regurgitant volume of ≥60 mL/beat [1].

Detailed chart reviews were performed on these patients (both alive and dead) to extract clinical and surgical data. The patients were divided into three groups: severe AS, severe AR, or mixed aortic valve disease, i.e., both AR and AS. 18 patients were identified for whom we employed the prophylactic use of an intra-aortic balloon pump (IABP). Patients who underwent other cardiac surgical procedures simultaneously were not excluded (27/40 patients).

Kaplan-Meier survival analysis (constructed in GraphPad Prism®, GraphPad Software, San Diego, California, USA) was also undertaken. An age and gender-matched control curve was constructed by matching each patient individually and following that patient according to life table survival data from the Centers for Disease Control and Prevention for the intercensal year 2010 (the mean year of operation). A single-sample log-rank test was employed for assessing the difference between patient and matched population survivals. The comparison of the operated group against the controls was constructed up to 5 years [12]. Further statistical analysis was conducted using the R programming language.

### 2.2 Operative Technique

All surgeries were performed via median sternotomy. Standard ascending aortic and dual stage venous cannulation were employed. Myocardial preservation was by a combination of systemic hypothermia, topical hypothermia with iced saline, and cold crystalloid cardioplegia given initially antegrade (except in case of severe AR) and subsequently retrograde through the coronary sinus. Cardiopulmonary bypass and cross-clamp times are recorded in the Results section. Intra-aortic balloon pump, when employed, was used prophylactically through the groin pre-bypass. The decision to use IABP was based on a gestalt of the following factors: how low the EF was; the degree of left ventricular enlargement; the overall appearance of the patient and how compromised they seemed; and the severity of the AR. IABP was used liberally in compromised patients, as it is the authors’ opinion that it provides strong perioperative cardiac support without the oxygen debt related to inotropes.

### 2.3 Study Endpoints

Primary endpoints for this study were changes in EF and mortality at 30 days and in the mid-term (beyond 1 year post-op). Changes in EF and LV dimensions over time were measured by echocardiography done during follow-up visits. These follow-ups were obtained regularly and at periodic intervals as post-operative visits or follow-ups in clinics. A typical follow-up consisted of clinical examination and serial echocardiography. Where direct patient follow-up was not possible, follow-up was obtained from the patient’s family physician.

### 2.4 Institutional Review Board

The study was approved by the Human Investigation Committee of Yale University. Individual patient consent was waived for retrospective review.

### 2.5 Preoperative Assessment

Preoperative clinical data, including chest *x*-ray, Doppler echocardiography, cardiac catheterization, and coronary artery anatomy, were collected by review of the medical records of 40 eligible patients, representing 4.47% of the entire population of patients who underwent SAVR for AS and/or AR (n = 895) by the single surgeon during the same period. All patients underwent two dimensional and Doppler echocardiography ≤30 days before the operation. LVEF was determined by a trained echocardiographer or cardiologist. Aortic valve hemodynamic data were calculated by standard methods and aortic valve area by the continuity equation. CAD was defined as ≥50% luminal diameter narrowing of the left main coronary artery or ≥70% narrowing of one or more major epicardial vessels and was in each case diagnosed as part of the normal cardiac work-up.

## 3. Results

From 2004 to 2019, 895 patients underwent SAVR for aortic stenosis, aortic regurgitation, or mixed disease by a single surgeon at our institution. 16 patients presented with AS, 20 with AR, and 4 with a combination of AS and AR. There were 35 men and 5 women, with a mean age of 63.7 at the time of operation. SAVR was most commonly performed either in isolation or in combination with an ascending aortic aneurysm resection (13 each), with a further 7 patients receiving concomitant CABG. Amongst the patients who had concomitant CABG, 3 had vein grafts only, 3 had an arterial graft only, while 4 had a mixture of both types of graft.

### 3.1 Echocardiographic Outcomes

**Fig. 1.**
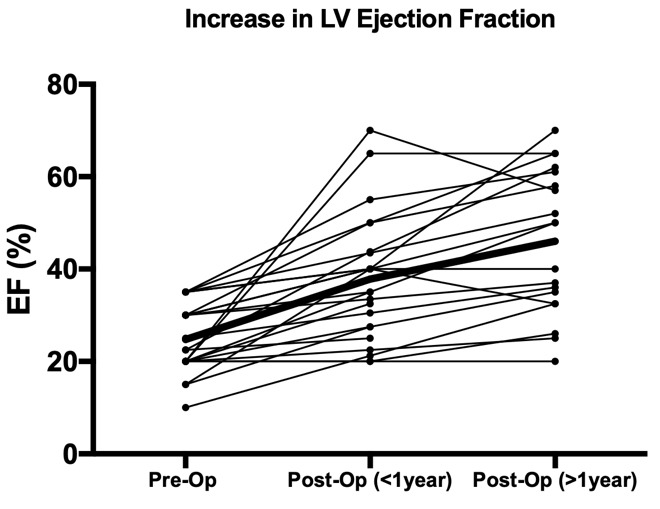
**Changes in EF over time following AVR**. One dot represents one patient’s reading, with lines representing changes over time for each patient. “Post-Op (>1 year)” refers to the most recent EF reading for each patient. Note marked, continued improvement post-surgery.

The mean ejection fraction improved from 26% to 35% in the first year and up to 46% beyond 1 year with mean follow-up of 43 months (0.1–140.7) when the most recent scan result was considered. These results are illustrated graphically in Fig. 1. The diagnosis sub-group with the lowest initial EF was the AS-only group (mean 24.8%), followed by AR-only group (mean 26.2%) and then the AS+AR group (mean 31.5%). The EF of all three subgroups improved significantly postoperatively when the most recent follow-up EFs were considered (unpaired two-tailed *t*-tests; +17.0% *p *< 0.001 for AS, +13.9% *p* = 0.002 for AR, and +15% *p* = 0.046 for AS and AR). There was no statistically significant difference in the initial EF (*p* = 0.26) or the change in EF (*p* = 0.714) between the three groups. For those who had simultaneous CABG, there was no significant difference in EF change between the three graft vessel choices (vein, artery, or both; *p* = 0.98) or between CABG patients and non-CABG patients (*p* = 0.69).

The prevalence of common cardiovascular risk factors, further operative details, and further echocardiogram readings for each group are listed in Table 1. Comparisons between the groups to assess for statistically significant differences were done using $\chi^{2}$ tests for categorical variables and one-way analysis over variance (ANOVA) for continuous variables. Regression analysis on the pre-operative operative variables in Table 1 shows that only smoking status independently predicts the increase in EF (*p* = 0.0282; +21.2% for never-smokers and +9.9% for others). The initial EF did not independently predict the change in EF (*p* = 0.38).

**Table 1.** **Summary of pre-operative risk factors, details of the operations, and pre-operative echocardiogram measurements**.

Variable		AR	AS	Mixed	Overall	Significance
Risk factors						
	Age at Surgery	56.9	70.6	69.5	63.6	*p* = 0.004*
	Height/m	1.78	1.72	1.82	1.76	*p* = 0.072
	Weight/kg	92.5	87.5	100.9	91.4	*p* = 0.389
	% Diabetes	10	50	50	30	*p* = 0.02*
	% COPD	10	18.75	0	12.5	*p* = 0.553
	% Hypertension	70	68.75	100	72.5	*p* = 0.429
	% Dyslipidemia	40	56.25	50	47.5	*p* = 0.621
	% Cancer	10	43.75	25	25	*p* = 0.067
	% Smokers	52.6	26.7	50	42.1	*p* = 0.296
	% Bicuspid aortic valve	45	25	25	35	*p* = 0.415
	EuroScoreII	6.3	10.1	5.8	7.8	*p* = 0.541
Operative details						
	Cross-clamp time/min	103	90	74	92	*p* = 0.227
	Cardiopulmonary bypass time/min	138	113	115	123	*p *= 0.345
	% Bioprosthetic valve	50	43.75	100	52.5	*p* = 0.125
Echocardiogram measurements						
	LV end-diastolic diameter (EDD)	6.5	5.5	6.1	6.0	*p* = 0.007*
	LVEDD <1 year follow-up	6.3	5.7	5.3	5.9	*p* = 0.312
	LVEDD >1 year follow-up	5.5	5.2	4.9	5.3	*p* = 0.466
	LV end-systolic diameter (ESD)	5.1	4.5	5.0	4.8	*p* = 0.18
	LVESD <1 year follow-up	5.0	4.6	4.5	4.8	*p* = 0.642
	LVESD >1 year follow-up	4.2	4.2	3.7	4.1	*p* = 0.666
	LV interventricular septum width (IVS)	1.1	1.1	1.3	1.1	*p* = 0.278
	LVIVS <1 year follow-up	1.1	1.1	1.2	1.1	*p* = 0.739
	LVIVS >1 year follow-up	1.1	1.0	1.2	1.1	*p* = 0.119
	LV posterior wall thickness (PW)	1.1	1.1	1.2	1.1	*p* = 0.75
	LVPW <1 year follow-up	1.1	1.1	1.2	1.1	*p* = 0.599
	LVPW >1 year follow-up	1.1	1.0	1.2	1.1	*p* = 0.133

Values quoted are percentages or mean values. “Significance” denotes whether the three groups (AS/AR/Mixed) are statistically different to each other based on $\chi^{2}$ tests for categorical variables and one-way ANOVA for continuous variables. * = Statistically significant (*p *< 0.05).

Patients in whom IABP was used had a lower initial EF than those without IABP (range 10–35, mean 23% with IABP vs. range 15–35%, mean 27.6% without IABP). This was a statistically significant difference (*p* = 0.014). Those who received IABP had a smaller EF increase (+12.3% vs. +19.0%), but this difference was not significant (*p* = 0.18).

In addition to EF, changes in LV dimensions were tracked over the same period. As shown in Table 1, in the mid-term, both LV end-diastolic diameter (LVEDD) and LV end-systolic diameter trended downwards (LVESD), while LV intraventricular septal thickness and posterior wall thickness remained relatively steady. Statistical analysis shows that only the decrease in LVEDD was significant over this period (*p* = 0.0046), though the decrease in LVESD over this period almost reached significance (*p* = 0.056). These results further demonstrate post-operative LV remodeling.

### 3.2 Mortality Outcomes

**Fig. 2.**
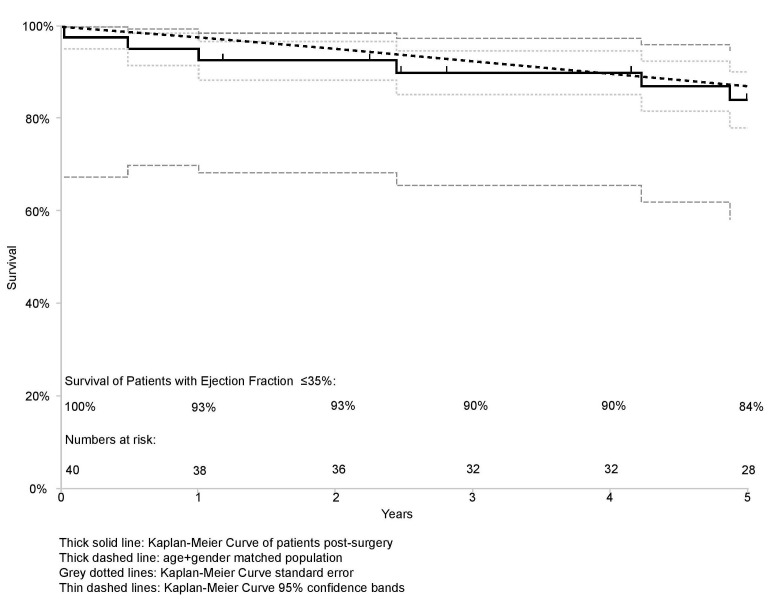
**Kaplan-Meier curves for the post-op and age/gender-matched control cohorts**. Note, remarkably, that survival of the low-EF surgical group equals that of the normal age and gender matched population.

Overall morbidity and mortality were low. A total of 9 patients (22.5%) died during the follow-up period (4 AS (25%), 4 AR (20%), and 1 mixed (25%)), with a mean of 1336 days between operation and death (range 10–2889 days). The mean days till death was 834 days in the AS-only group, 1728 days in the AR-only group, and was 1778 days in the single mixed patient. Only 2 patients died from a cardiovascular cause. Five-year survival was comparable between the study group and an age- and gender-matched general population (*p* = 0.834), as seen in Fig. 2.

Only one patient, a male who was 47 at the time of the operation, died within 30 days of the operation. He had AR only and received concomitant ascending aortic aneurysm replacement. Two days after discharge, he developed chest pain, tightness, and shortness of breath, and had to be readmitted to hospital. He was found to have a large LAD thrombus, requiring single CABG to the LAD and LVAD insertion. Due to a lack of improvement from his state of cardiogenic shock, care was withdrawn in the ICU two days later, a total of 10 days post-op.

Of the pre-operative and operative variables outlined in Table 1, only older age (*p* = 0. 0195; OR 1.10 (1.03-1.21); mean 60.8 for alive and 73.8 for dead) and higher EuroScore II (*p* = 0.0375; OR 1.17 (1.04–1.41); mean 5.1 for alive and 16.8 for dead) independently predicted mid-term mortality. The change in EF also predicted mid-term mortality (+18.9% alive vs. +5.7% dead, *p* = 0.037), but importantly, the initial EF did not (*p* = 0.45).

Two of the patients who died had AVR only. The remainder had AVR + CABG (3), AVR + aneurysm repair (3), or AVR + CABG + aneurysm repair (1). There was no significant difference in mid-term mortality between the three choices of vessel graft (vein, artery, or both; *p* = 0.91). The distribution of operation type in the death subset was not significantly different from the distribution in all patients taken together ($\chi^{2}$ test *p* = 0.78).

In addition to mortality, the incidence of other major cardiovascular events- post-op MI and stroke- were tracked. Post-op MI occurred in 5.1% of cases (AR: 10%, AS + Mixed: 0%) and post-op stroke occurred in 10.3% of cases (AR: 15%, AS: 6.7%, Mixed: 0%). ANOVA tests reveal there is no significant difference between the three groups for either of these two outcomes (*p* = 0.37 for MI, *p* = 0.56 for stroke).

## 4. Discussion

We report our results with SAVR in patients with an impaired left ventricle, who had AS, AR, or both. We show that early and late post-surgical outcomes are excellent. We show that even in patients with severely impaired left ventricles, i.e., EF <35%, AVR can be performed safely and improves patient outcomes as shown by an increase in EF and an improvement in LV dimensions. Age and EuroScore II should be considered when predicting patient mortality, while smoking status most strongly predicts the degree to which the patient’s EF can be expected to improve. The initial EF did not independently predict the change in EF or mortality in this cohort. These results support the growing body of recent work which illustrates the positive utility of AVR in low-EF patients with AS [13] or AR [14, 15], but with a specific focus on SAVR. It is perhaps surprising that in recent years, the valve intervention rate has not been increasing as quickly as might be expected given the mounting evidence of the efficacy and safety of these valve procedures [4].

From the perspective of functional outcomes, the predictive value of smoking status is in line with many prior studies which illustrate the link between smoking and LV dysfunction, including a recent study that used UK Biobank data to show how this association persists even when controlling for other common risk factors such as hypertension and diabetes mellitus [16]. The effect may even be dose-dependent in ex-smokers [17]. The etiology of this relationship is thought to be multifactorial, involving inflammatory and oxidative damage to the vascular endothelium and subsequent ischaemic damage to the myocardium [16, 18]. There may even be damage to the myocardium directly [19, 20]. *In-vitro* studies using cigarette smoke extract have also shown that smoke can damage cardiac stem cells, which in turn would impair the repair of cardiac injury [21].

Reports of short-term mortality of patients who underwent SAVR for AS and/or AR with low EF vary widely in the literature, from as low as 0% to a high of 21%. A number of variables have been proposed to predict prognosis in patients, some of which are different from those identified in the present study [22, 23, 24]. Kaneko *et al*. [14] found that advanced age, high NYHA class, and elevated preoperative creatinine were associated with an increased risk of mortality in their cohort. Pereira *et al*. [25] showed that age and serum creatinine level were predictors of mortality in patients who received AVR for severe AS with low transvalvular gradient and severe left ventricular dysfunction (EF ≤35%). Four-year survival in the AVR group was 78%, compared to just 15% for non-operated controls [25]. Clavel *et al*. [26] found that older age, NYHA class III/IV, atrial fibrillation, chronic kidney failure, diabetes, coronary artery disease, Duke myocardial jeopardy score, chronic obstructive pulmonary disease, higher LV mass index, and lower peak aortic jet velocity were significant predictors of poor midterm survival in patients who underwent AVR for severe AS.

Bach *et al*. [27] concluded that some patients with severe symptomatic AS were denied access to potentially lifesaving therapy due to a perception of prohibitive operative risk. They found that 191 (52%) patients with severe AS at their centers did not undergo AVR, of which 33 (17%) had an EF <50%. Of those not operated on, 126 (66%) had symptomatic AS, and 61 (48%) of these symptomatic patients were not operated on due to comorbidities and operative risk. Though a low EF was not explicitly mentioned here, it has previously been shown to be amongst the strongest individual predictors of denying surgery [28]. Furthermore, the Society of Thoracic Surgery Adult Cardiac Surgery Risk Calculator, which was used in the study to calculate operative risk, considers heart failure as a contributing factor to poor outcomes. Though decisions will depend on the individual clinician and institution, it is still clear that a low EF will factor into the risk equation to some extent. The results of the present study better illustrate the true extent of this risk, finding risk to be quite low in this experience.

In addition to SAVR, other operations such as CABG have also been investigated in the low EF population. Such studies similarly illustrate that low EF need not preclude an operation as long-term survival is the same or better when compared to non-operated controls. As with SAVR, an increase in EF is also commonly seen, and this may contribute to improving any associated ventricular dysfunction. A study from 2001, for example, followed 135 patients with an EF <35% who were treated with CABG. Post-op follow-ups revealed an average long-term EF increase of 10% and resolution or near-complete resolution of angina [29]. The operation was therefore beneficial from both a quality of life and a long-term survival perspective. Another study from 1997 showed long-term survival for CABG patients with a pre-op EF of 10–20% was similar to those with a higher EF of 21 to 30%, and even depressed right ventricular function did not statistically impact long-term survival [30]. Taken together, these data provide more reassurance to surgeons intending on operating on low EF patients. These data manifest the considerable potential for myocardial recovery upon revascularization or relief of outflow obstruction intrinsic to AS.

### Limitations

Our study has several important limitations. These include its retrospective nature and the observational nature of the study design. Moreover, the small number of patients may have limited the power of the study to detect clinically important differences between groups. It also remains to be seen whether the results are generalizable.

## 5. Conclusions

A regurgitant or stenotic aortic valve can contribute to progressive LV dysfunction (LVD) through an increase in afterload or a volume overload. Conceptually, if a patient’s LV dysfunction were caused at least in part by their valve lesion, then it may not be prudent to dismiss the patient as a surgical candidate based primarily on their LVD, as we might expect it to improve following correction of the valve dysfunction. The data from this study suggest that this theory is borne out: the EF improved on average by 77% above the pre-operative value, while 5-year survival was rendered comparable to an age- and gender-matched population. As noted in previous work from our group, there are a few factors which cumulatively contribute to the risks of this operation: the severity of pre-op symptoms, the severity of the left ventricular systolic dysfunction, and the duration of this dysfunction [31]. Operating in a timely manner may thus prevent long-term, irreversible LV dysfunction from developing.

Our data suggest that left ventricular failure, specifically manifesting as EF ≤35%, need not preclude an AVR operation in patients with severe AS or AR. These findings will add support to current AHA guidelines and will add to surgeon confidence when making the decision to perform surgery in this jeopardized subset of patients.
